# Initiation and promotion at different ages and doses in 2200 mice. II. Decrease in promotion by TPA with ageing.

**DOI:** 10.1038/bjc.1981.142

**Published:** 1981-07

**Authors:** F. Stenbäck, R. Peto, P. Shubik

## Abstract

Using the data described in Paper I, we compare the effects of the same treatment timings and doses given at different ages. Initiation with DMBA at 68 weeks of age, followed 3 weeks later by TPA, has a significantly (P less than 0.0001) less rapid effect on subsequent tumour incidence than does initiation at 8 or at 48 weeks of age, followed 3 weeks later by TPA. We suggested that this is chiefly due not to changes in the numbers of cells initiated by DMBA, but rather to a decrease in the promotional efficacy of TPA in ageing mice.


*Br. J. Cancer* (1981) **44**, 15

### INITIATION AND PROMOTION AT DIFFERENT AGES AND DOSES IN 2200 MICE
### II. DECREASE IN PROMOTION BY TPA WITH AGEING

F. STENBÄCK*, R. PETO† AND P. SHUBIK‡

*From the Eppley Institute for Cancer Research, Omaha, Nebraska, U.S.A.*

Received 29 April 1980 Accepted 27 March 1981

**Summary.—Using the data described in Paper I, we compare the effects of the same treatment timings and doses given at different ages. Initiation with DMBA at 68 weeks of age, followed 3 weeks later by TPA, has a significantly ($P < 0{\cdot}0001$) *less* rapid effect on subsequent tumour incidence than does initiation at 8 or at 48 weeks of age, followed 3 weeks later by TPA. We suggest that this is chiefly due not to changes in the numbers of cells initiated by DMBA, but rather to a decrease in the promotional efficacy of TPA in ageing mice.**

MANY qualitatively different processes have been found to affect cancer induction, and the most promising framework for an eventual synthesis of these several different mechanisms into a coherent description of the natural history of cancer is, especially for epithelial tumours, some kind of multi-stage model. One simplifying assumption commonly made when formulating multi-stage models (*e.g.* Armitage & Doll, 1961; Peto, 1977; Whittemore & Keller, 1978) is that age *per se* has little or no intrinsic relevance to the processes of cancer induction.§ This assumption predicts that, for a particular carcinogenic treatment which strongly affects, *inter alia*, the *first* stage of cancer induction, the cancer risk within a fixed time after that treatment began should not, in general, depend strongly on how old the animal was when the treatment began (excluding the peculiarly vulnerable foetal and neonatal periods). This is an easy prediction to test experimentally but, despite this, there is surprisingly little published evidence available about the effects of age on the carcinogenicity of treatments which include some initiation. The most direct experimental evidence (and a review of other experimental and epidemiological evidence) is perhaps that given by Peto *et al.* (1975), who found that when mice were given 20 $\mu$g of benzpyrene twice weekly, starting at ages 10, 25, 40 or 55 weeks of age and continuing indefinitely thereafter, the resultant cancer risk was independent of age. The risk depended strongly on how long the treatment had been given, of course, but among animals of different ages which had all been treated for the same length of time the cancer incidence rate did not depend on age.

This experiment yielded exactly the result predicted by the simplest multistage model theory; the cumulative result

* Requests for reprints to: Department of Pathology, University of Oulu, SF 90220, Oulu 22, Finland.

† Imperial Cancer Research Fund, Reader in Cancer Studies, Nuffield Department of Clinical Medicine, Radcliffe Infirmary, Oxford OX2 6HE.

‡ Imperial Cancer Research Fund Cancer Epidemiology & Clinical Trials Unit, Oxford OX1 3QG.

§ If, for example, a cell must undergo 4 age-independent changes before becoming neoplastic, then the probability of all 4 happening before age 60 might be $2 \times 2 \times 2 \times 2 = 16$ times greater than the probability of all 4 happening before age 30, so the independence of age of the separate components of the whole process of cancer induction does provide a natural explanation for the well known increase of cancer risks with increasing duration of accumulation of damage.

of the sequence of processes which are presumably involved in cancer induction seemed to be unaffected by the age of the animals. However, if the first stage was easier to induce in older animals and some later stage was more difficult to induce, these two effects could, in principle, approximately cancel out, leaving the final cancer incidence rate largely independent of age (among animals treated for the same duration), as observed. The present study examines the dependence on age at initiation in a system which, unlike that studied by Peto *et al.* (1975) does involve specific promotional stimuli (due either to wound healing or to use of a promoting agent) and some quite marked dependences now emerge. If the ease with which promotion can operate does vary markedly with age, all attempts to make a "multi-stage" synthesis of what is known about initiation and promotion will be seriously incomplete unless this age dependence is allowed for.

### METHODS

Among the 9 treatment groups of the initiation/promotion experiment described in the accompanying paper (Stenbäck *et al.*, 1981) there are some groups which have the same interval between initiation and the start of promotion, and which differ only in the age of the animals during treatment. These groups, and the analyses which we shall perform on them, are listed in the Table. We can assess separately the dependence on age at treatment of the effects of (a) initiation followed by immediate promotion, (b) initiation followed by delayed promotion, and (c) initiation without promotion.

In each case, we may assess the response either in terms of (i) the *total number of tumours* arising within a particular 20-week period of time (to which one mouse may contribute more than one tumour, which makes the calculation of reliable *P* values difficult), or in terms of (ii–iv) the *number of tumour-bearing animals*, *i.e.* by a standard time-to-first-tumour analysis, with *P* values, of (ii) all tumours, of any size or type, or (iii) 10mm tumours, or (iv) malignant tumours.

Details of the experimental and statistical methods are given in the previous paper by Stenbäck *et al.* (1981).

TABLE.—*Times of initiation and promotion in various subgroups of animals.* Each group was replicated with 10, 30, 100 or 300 μg of DMBA as the initiating dose.

| Group | Age (weeks at initiation) | Ages during promotion | Weeks between initiation and promotion |
|---|---|---|---|
| Initiation followed by immediate promotion | | | |
| a | 8 | 11–26 | 3 |
| f | 48 | 51–66 | 3 |
| h | 68 | 71–86 | 3 |
| Analysis: from Week 4 from initiation. | | | |
| Initiation followed by delayed promotion, ignoring tumours which arise before promotion | | | |
| c | 8 | 31–46 | 23 |
| g | 48 | 71–86 | 23 |
| Analysis: from Week 24 from initiation. | | | |
| 23 unpromoted weeks after initiation (except for the high-dose animals, who underwent promotion by wound healing as ulcers and erosions caused by the initiator recovered) | | | |
| { c | { 8 | { 31– | 23 |
| d | 8 | 51– | 43 |
| e | 8 | 71– | 63 |
| i } | 8 } | never } | — |
| g | 48 | 71– | 23 |
| Analysis: during Weeks 4–23 from initiation only with the bracketed groups pooled and treated as one group. | | | |

### RESULTS

### *The dependence on age of the effects of initiation followed by immediate promotion*

Initiation at age 8 weeks followed by immediate promotion, and initiation at 48 weeks followed by immediate promotion, had similar effects on the total numbers of tumour-bearing animals (Fig. 1: for numerical details, see Appendix Tables a and b). But, for every index of response, initiation at 68 weeks followed by immediate promotion had much less effect. The relative diminution of effect was greater when assessed in terms of large or of malignant tumours than when assessed in terms of papillomas, but even for time to first tumour the shortfall was highly significant ($P < 0{\cdot}0001$). This dimi-

Fig. 1.—Tumour response: O/E values comparing Groups a, f, and h, initiated at 8, 48 and 68 weeks and promoted 3–18 weeks later (from Appendix Tables a and b). ● All tumours within 20 weeks. ○ First tumours of any size or type (with approximate 95% confidence intervals). □ First 10mm tumours. △ First malignant tumour.

Fig. 2.—Tumour response: O/E values comparing the responses to delayed promotion in Groups c and g, initiated at 8 and 48 weeks, respectively and promoted 23–38 weeks later (from Appendix Tables c and d). Symbols as in Fig. 1.

nution of effect is confirmed by the life-table analysis provided by van Duuren *et al.* (1978) in which it was shown that initiation at Week 6 of age followed by promotion from Week 8 onwards produced papillomas more rapidly than initiation at Week 56 followed by promotion from Week 58 onwards.

Our data indicate that at between 50 and 80 weeks of age there is a substantial decrease either in the response to DMBA or in the response to TPA (which was applied to Group h from Weeks 71 to 86), and shows that multistage models must be formulated with great care in the particular context of initiation and promotion.

*The dependence on age of the effects of initiation followed by delayed promotion*

For all 4 indices of response there was a slightly but non-significantly lower response to promotion among animals initiated at Week 48 and promoted at Weeks 71–86 than in animals treated 40 weeks earlier (Fig. 2: for numerical details, see Appendix Tables c and d). The decrease does not seem as marked as in Fig. 1, where initiation at Week 68 and promotion at Weeks 71–86 were studied, even though the age at promotion was the same.

*The dependence on age of the short-term effects of initiation without immediate promotion*

Surprisingly, for DMBA given alone with no immediate promotion, there appears to be clear evidence of an *increased* short-term yield of tumours if initiation is at 48 rather than 8 weeks of age, tumours being observed during the subsequent 23 weeks without promotion (Fig. 3: for numerical details, see Appendix Tables e and f). This is odd, since it concerns an agent rather similar to that studied by Peto *et al.* (1975) with which no age-related changes in effect were evident (and see also the footnote to Appendix Table e), but it is too highly significant a finding to be dismissed easily as the result of chance. It is argued in Stenbäck *et al.* (1981) that if the effect is real, the most plausible explanation is that the healing of ulcers, erosions and related changes is slower in old mice than in young ones, and that the promotional stimulus associated with such healing is therefore more pro-

FIG. 3.—Tumour response: observed ÷ expected numbers. O/E values for the groups for the response to initiation alone of Groups c and g, initiated at 8 and 48 weeks and observed during the next 4–23 weeks (from Appendix Tables e and f). Symbols as in Fig. 1.

longed and so more potent. Whatever the explanation for this increase in the effects of DMBA *alone*, it indicates that, in order to produce the rather marked decreases in the effects of initiation *with* promotion that was seen in Fig. 1, there must be an even stronger age-related decrease in promotional effects of TPA than was suggested by Fig. 1 alone.

(It may be noteworthy that the total number of tumours arising during 23 weeks without applied promoter plus 20 weeks containing a period of applied promoter is about the same for initiation at 8 as for initiation at 48 weeks. It is just that at 48 weeks we get more tumours without promotion and then fewer during promotion. This is, however, perhaps coincidental rather than significant.)

### DISCUSSION

Pereira *et al.* (1979) have measured directly the amount of benzpyrene which actually binds to mouse epidermal DNA, and have found simple proportionality between the amount applied and the amount subsequently bound to the DNA. If the same can be done for DMBA then it will be possible, by studying animals of different ages, to determine whether age affects the proportion of an applied dose of DMBA which eventually binds to the DNA. Likewise, certain biochemical effects of TPA applied to the skin of living mice, such as ornithine decarboxylase induction, are quantitatively measurable (Verma *et al.*, 1978), as are certain histological effects (Klein-Szanto *et al.*, 1980) and it would therefore be possible to determine whether the age of a mouse affects the biochemical or histological response of its skin to TPA. Specific measurements such as these may yield more direct evidence than the present experiment does as to whether it is initiation or promotion which takes place less readily in older animals.

There is a large literature on mathematical formulations of multi-stage models (for review and references, see Whittemore & Keller, 1978), almost all of which implicity or explicitly assumes that some or all of the transitions through which cells pass *en route* from normality to full transformation are largely independent of age. Our data show clearly that such assumptions are not valid for the special case of initiation with a single potent dose of DMBA followed by promotion by wound healing or by TPA. This does not prove that such assumptions are also seriously wrong under the conditions of chronic exposure to much lower environmental doses of various chemicals (or to spontaneous cellular accident) that are usually more relevant to human carcinogenesis. However, it does suggest a need for more caution in the mathematical formulation of multi-stage models than has commonly been exercised. Our results do not cast any doubt on the fundamental assumption of multi-stage model theory, that accumulation of more than one heritable change in a normal tissue cell is needed before that cell can act as the progenitor of a neoplastic clone. They do, however, indicate that many of the quantitative formulations of the consequences of this fundamental assumption may have been seriously over-simplified, at least where they deal with specific promotional stimuli.

Initiation with toxic or near-toxic doses of DMBA followed by strong promotion is obviously a poor model for the very slow accumulation of precancerous changes in human tissues, and the aetiology of papillomas may obviously be a very poor model for that of infiltrating carcinomas. However, the anomalies we have discovered suggest that until the nature of the various stages of human carcinogenesis is understood and their rates of occurrence under realistic conditions of mild insult can be directly determined, the formulation of quantitative models for multistage carcinogenesis should be approached more cautiously that hitherto.

### REFERENCES

ARMITAGE, P. & DOLL, R. (1961) Stochastic models for carcinogenesis. In *Proc. 4th Berkeley Symposium on Mathematical Statistics and Probability: Biology and Problems of Health*, 4. Berkeley: University of California Press. p. 19.

INTERNATIONAL AGENCY FOR RESEARCH ON CANCER (1980) Guidelines for simple, sensitive significance tests for carcinogenic effects in long-term animal experiments. In *IARC Monographs on the Evaluation of the Carcinogenic Risk of Chemicals to Humans*, Suppl. 2. Ed. Montesano. Lyon: I.A.R.C. p. 311.

KLEIN-SZANTO, A. J. P., MAJOR, S. K. & SLAGA, T. J. (1980) Induction of dark keratinocytes by 12-O-tetradecanoyl-phorbol-13-acetate and mezerein as an indicator of tumor-promoting efficiency. *Carcinogenesis*, **1**, 399.

PEREIRA, M. A., BURNS, F. J. & ALBERT, R. E. (1979) Dose response for benzo(a)pyrene adducts in mouse epidermal DNA. *Cancer Res.*, **39**, 2556.

PETO, R. (1977) Epidemiology, multistage models and short-term mutagenicity tests. In *Origins of Human Cancer*. Ed. Hiatt *et al.* New York: Cold Spring Harbor Laboratory. p. 1403.

PETO, R., ROE, F. J. C., LEE, P. N., LEVY, L. & CLACK, J. C. (1975) Cancer and ageing in mice and men. *Br. J. Cancer*, **32**, 411.

STENBÄCK, F., PETO, R. & SHUBIK, P. (1981) Initiation and promotion at different ages and doses in 2200 mice. I. Methods, and the apparent persistence of initiated cells. *Br. J. Cancer*, **44**, 1.

VAN DUUREN, B. L., SMITH, A. C. & MELCHIONNE, S. M. (1978) Effect of aging in two-stage carcinogenesis on mouse skin with phorbol myristate acetate as promoting agent. *Cancer Res.*, **38**, 865.

VERMA, A. K., RICE, H. M., SHAPAS, B. G. & BOUTWELL, R. K. (1978) Inhibition of 12-O-tetradecanoyl-phorbol-13-acetate-induced ornithine decarboxylase activity in mouse epidermis by vitamin A analogs (retinoids). *Cancer Res.*, **38**, 793.

WHITTEMORE, A. & KELLER, J. (1978) Quantitative theories of carcinogenesis. *S.I.A.M. Rev.*, **20**, 1.

### Appendix

### Table a.—*The effects of age on total tumour yield after immediate promotion*

Total numbers of tumours (including second and subsequent tumours) during the 20 weeks from the start of promotion, among animals initiated at different ages, promoted during Weeks 3–18 thereafter, and surviving at least 23 weeks after initiation.

MS = Number of Mice Surviving at least 23 weeks after initiation.

O = Number of tumours Observed to arise on these survivors during Weeks 4–23.

E = Number of tumours Expected to do so if the number per survivor depends on dose level but not on age at initiation.

T/M = Tumours per mouse—O/MS.

| Protocol | a | | | f | | | h | | | | | |
|---|---|---|---|---|---|---|---|---|---|---|---|---|
| Age at initiation | 8 | | | 48 | | | 68 | | | Totals | | |
| Age during promotion | 11–26 | | | 51–66 | | | 71–86 | | | (3 protocols) | | |
| | MS | O | E | MS | O | E | MS | O | E | MS | O | E |
| 300 μg initiation | 73 | 146 | 117·9 | 40 | 55 | 64·6 | 30 | 30 | 48·5 | 143 | 231 | 231·0 |
| | 2·0 T/M | | | 1·4 T/M | | | 1·0 T/M | | | 1·6 T/M | | |
| 100 μg initiation | 34 | 49 | 33·1 | 37 | 39 | 36·0 | 38 | 18 | 36·9 | 109 | 106 | 106·0 |
| | 1·4 T/M | | | 1·1 T/M | | | 0·5 T/M | | | 1·0 T/M | | |
| 30 μg initiation | 38 | 25 | 30·9 | 47 | 47 | 38·2 | 38 | 28 | 30·9 | 123 | 100 | 100·0 |
| | 0·7 T/M | | | 1·0 T/M | | | 0·7 T/M | | | 0·8 T/M | | |
| 10 μg initiation | 39 | 25 | 19·3 | 52 | 33 | 25·8 | 36 | 5 | 17·9 | 127 | 63 | 63·0 |
| | 0·6 T/M | | | 0·6 T/M | | | 0·14 T/M | | | 0·5 T/M | | |
| Total of above (all doses) | 184 | 245 | 201·2 | 176 | 174 | 164·6 | 142 | 81 | 134·2 | 502 | 500 | 500·0 |
| Total O ÷ total E* | O/E = 1·22 | | | O/E = 1·06 | | | O/E = 0·60 | | | O/E = 1·00 necessarily | | |

* It is not valid to compare the average number of tumours per mouse in the total for all doses, because the proportion of high-dose animals in Protocol a is greater than in Protocol h. However, the ratios of Total O to Total E can be compared validly with each other, as here.

TABLE b.—*The effects of age on the number of tumour-bearing animals, after immediate promotion*

Incidence rates of (ii) first tumours irrespective of size or type, (iii) first 10 mm tumours, and (iv) first malignant tumours, among animals initiated at various ages and promoted from Weeks 3–18 thereafter. The expected numbers, E, and $P$ values were calculated using methods of analysis appropriate for *in vivo* tumours, using the times when (ii) appearance, (iii) size > 10 mm and (iv) apparent malignancy were first noted.

N = Number of animals alive at the end of Week 3 after initiation, excluding any which had already developed the tumour type of interest.

O = Number of such animals which were Observed to develop the tumour type of interest at any time after Week 3.

E = Number of such animals Expected to do so if onset rates depend on dose level and on time since initiation but not on age at initiation. These Expecteds were calculated using the methods described for tumours observed in a mortality-independent context in IARC (1980).

| | | Protocol a | | | Protoclo f | | | Protocol h | | | Totals (all 3 protocols) and $P$ values for negative trend, a→f→h | | |
|---|---|---|---|---|---|---|---|---|---|---|---|---|---|
| | | Initiation at age 8 weeks | | | Initiation at age 48 weeks | | | Initiation at age 68 weeks | | | | | |
| | | Promotion at 11–26 weeks | | | Promotion at 51–66 weeks | | | Promotion at 71–86 weeks | | | | | |
| Tumour type of interest | DMBA dose | N | O | E | N | O | E | N | O | E | N | O | E |
| (ii) any | 300 μg | 80 | 59 | 47·2 | 58 | 32 | 33·6 | 78 | 32 | 42·2 | 216 | 123 | 123·0 |
| (ii) any | 100 μg | 40 | 26 | 15·8 | 58 | 32 | 26·5 | 72 | 17 | 32·7 | 170 | 75 | 75·0 |
| (ii) any | 30 μg | 39 | 12 | 15·4 | 59 | 26 | 16·0 | 77 | 12 | 18·6 | 175 | 50 | 50·0 |
| (ii) any | 10 μg | 40 | 14 | 10·8 | 56 | 23 | 14·9 | 70 | 5 | 16·3 | 166 | 42 | 42·0 |
| (ii) any tumours | all* doses | 199 | 111 | 89·2 | 231 | 113 | 90·9 | 297 | 66 | 109·9 | 727 | 290 | 290·0 |
| Total O ÷ total E | | O/E = 1·24 | | | O/E = 1·24 | | | O/E = 0·60 | | | $P < 0{\cdot}0001$ | | |
| (iii) 10mm tumours | all* doses | 199 | 61 | 50·3 | 232 | 44 | 32·2 | 303 | 2 | 24·5 | 734 | 107 | 107·0 |
| Total O ÷ total E | | O/E = 1·21 | | | O/E = 1·37 | | | O/E = 0·08 | | | $P < 0{\cdot}0001$ | | |
| (iv) malignant tumours | all* doses | 199 | 40 | 34·9 | 232 | 26 | 20·6 | 303 | 3 | 13·5 | 734 | 69 | 69·0 |
| Total O ÷ total E | | O/E = 1·15 | | | O/E = 1·26 | | | O/E = 0·22 | | | $P < 0{\cdot}05$ | | |

* These total N, total O and total E values are derived by summation of the N, O and E values in 4 separate dose-specific analyses.

TABLE c.—*The effects of age on total tumour yields after delayed promotion*

Total numbers of tumours (including second and subsequent tumours) during the 20 weeks from the start of promotion, among animals initiated at the start of promotion, among animals initiated at different ages, promoted during Weeks 23–38 thereafter, and surviving at least 43 weeks after initiation.

| | Protocol c: Initiation at age 8 weeks | | | Protocol g: Initiation at age 48 weeks | | | Total of c and g | | |
|---|---|---|---|---|---|---|---|---|---|
| | MS | O | E | MS | O | E | MS | O | E |
| 300 $\mu$g initiation | 34 | 27 | 27·2 | 11 | 9 | 8·8 | 45 | 36 | 36·0 |
| | 0·8 T/M | | | 0·8 T/M | | | 0·8 T/M | | |
| 100 $\mu$g initiation | 25 | 11 | 14·0 | 25 | 17 | 14·0 | 50 | 28 | 28·0 |
| | 0·4 T/M | | | 0·7 T/M | | | 0·6 T/M | | |
| 30 $\mu$g initiation | 32 | 18 | 14·6 | 36 | 13 | 16·4 | 68 | 31 | 31·0 |
| | 0·6 T/M | | | 0·4 T/M | | | 0·5 T/M | | |
| 10 $\mu$g initiation | 35 | 28 | 17·7 | 36 | 8 | 18·3 | 71 | 36 | 36·0 |
| | 0·8 T/M | | | 0·2 T/M | | | 0·5 T/M | | |
| Total of above (all doses) | 126 | 84 | 73·5 | 108 | 47 | 57·5 | 234 | 131 | 131·0 |
| Total O ÷ total E* | O/E = 1·14 | | | O/E = 0·82 | | | O/E = 1·00 necessarily | | |

* Footnote and abbreviations as in Table a.

TABLE d.—*The effects of age at initiation on the numbers of tumour-bearing animals after delayed promotion*

Incidence rate among animals initiated at various ages, alive and free of the tumour type of interest 23 weeks after initiation, and promoted from Week 23 after initiation for 15 weeks (or less for animals dying before Week 38). Notation as in Table b. The period of observation runs from Week 23 after initiation to death or to the first occurrence of a tumour of the type that is of interest.

| Tumour type of interest | DMBA dose | Protocol c: Initiation at 8 weeks | | | Protocol g: Initiation at 48 weeks | | | Total (both protocols) and *P* values for differences between c and g | | |
|---|---|---|---|---|---|---|---|---|---|---|
| | | N | O | E | N | O | E | N | O | E |
| (ii) any | 300 $\mu$g | 46 | 21 | 20·2 | 24 | 7 | 7·8 | 70 | 28 | 28·0 |
| (ii) any | 100 $\mu$g | 27 | 10 | 8·8 | 50 | 10 | 11·2 | 77 | 20 | 20·0 |
| (ii) any | 30 $\mu$g | 37 | 9 | 11·6 | 58 | 15 | 12·4 | 95 | 24 | 24·0 |
| (ii) any | 10 $\mu$g | 39 | 21 | 12·1 | 67 | 9 | 17·9 | 106 | 30 | 30·0 |
| (ii) any tumours | all* doses | 149 | 61 | 52·7 | 179 | 41 | 49·3 | 348 | 102 | 102·0 |
| Total O ÷ total E | | O/E = 1·16 | | | O/E = 0·83 | | | $P = 0{\cdot}07$ | | |
| (iii) 10mm tumours | all* doses | 178 | 25 | 23·1 | 232 | 12 | 13·9 | 410 | 37 | 37·0 |
| Total O ÷ total E | | O/E = 1·08 | | | O/E = 0·86 | | | $P = 0{\cdot}5$ | | |
| (iv) malignant tumours | all* doses | 182 | 19 | 18·0 | 242 | 8 | 9·0 | 424 | 27 | 27·0 |
| Total O ÷ total E | | O/E = 1·06 | | | O/E = 0·89 | | | $P = 0{\cdot}7$ | | |

* Footnote and abbreviations as in Table b.

TABLE e.—*The effects of age at initiation on the total tumour yield without TPA promotion*

Total numbers of tumours (including second and subsequent tumours) arising during Weeks 4–23 after initiation among animals initiated at different ages and alive and umpromoted for at least 23 weeks thereafter.

| | Protocols c, d, e and i: Initiation at age 8 weeks | | | Protocol g: Initiation at age 48 weeks* | | | Total of c, d, e, i and g | | |
|---|---|---|---|---|---|---|---|---|---|
| | MS | O | E | MS | O | E | MS | O | E |
| 300 μg initiation | 221 | 94 | 112·1 | 55 | 46 | 27·9 | 276 | 140 | 140·0 |
| | | 0·4 T/M | | | 0·8 T/M | | | 0·5 T/M | |
| 100 μg initiation | 211 | 66 | 59·4 | 59 | 10 | 16·6 | 270 | 76 | 76·0 |
| | | 0·3 T/M | | | 0·2 T/M | | | 0·3 T/M | |
| 30 μg initiation | 210 | 0 | 4·6 | 63 | 6 | 1·4 | 273 | 6 | 6·0 |
| | | 0·00 T/M | | | 0·10 T/M | | | 0·02 T/M | |
| 10 μg initiation | 214 | 0 | 1·5 | 72 | 2 | 0·5 | 286 | 2 | 2·0 |
| | | 0·00 T/M | | | 0·03 T/M | | | 0·01 T/M | |
| Total of above (all doses) | 856 | 160 | 177·6 | 249 | 64 | 46·4 | 1105 | 224 | 224·0 |
| Total O ÷ total E† | | O/E = 0·90 | | | O/E = 1·38 | | | O/E = 1·00 necessarily | |

* Tumour yield at 48 weeks. As part of a more recent experiment on animals of the same sex and strain, albeit from a different source, initiated similarly at 48 weeks with these same doses, the yields 23 weeks later were 4/40, 4/37, 0/43 and 0/40 tumours/mouse, suggesting that the figure of 0·8 T/M in this table may not be reproducible.

† Footnote and abbreviations as in Table a.

TABLE f.—*The effects of age at initiation on the numbers of tumour-bearing animals, without TPA promotion*

Incidence rates of (ii) first tumours, irrespective of size or type, (iii) first 10mm tumours, and (iv) first malignant tumours, among animals initiated at various ages, alive 3 weeks later and left for more than 3 weeks without promotion. The period of observations runs from 3 weeks after initiation to the eventual start of promotion, to death, or to the first onset of the tumour type of interest.

| Tumour type of interest | DMBA dose | Protocols c, d, e and i: Initiation at 8 weeks | | | Protocol g: Initiation at 48 weeks | | | Totals (both protocols) and *P* values for differences between 8 and 48 | | |
|---|---|---|---|---|---|---|---|---|---|---|
| | | N | O | E | N | O | E | N | O | E |
| (ii) any | 300 μg | 340 | 102 | 117·3 | 80 | 38 | 22·7 | 420 | 140 | 140·0 |
| (ii) any | 100 μg | 260 | 57 | 55·2 | 80 | 15 | 16·8 | 340 | 72 | 72·0 |
| (ii) any | 30 μg | 260 | 0 | 3·8 | 80 | 5 | 1·2 | 340 | 5 | 5·0 |
| (ii) any | 10 μg | 260 | 0 | 0·7 | 80 | 1 | 0·3 | 340 | 1 | 1·0 |
| (ii) any tumours | all* doses | 1120 | 159 | 177·1 | 320 | 59 | 40·9 | 1440 | 218 | 218·0 |
| Total O ÷ total E | | | O/E = 0·90 | | | O/E = 1·44 | | | $P < 0·002$ | |
| (iii) 10mm tumours | all* doses | 1120 | 29 | 36·6 | 320 | 17 | 9·4 | 1440 | 46 | 46·0 |
| Total O ÷ total E | | | O/E = 0·79 | | | O/E = 1·82 | | | $P < 0·01$ | |
| (iv) malignant tumours | all* doses | 1120 | 6 | 10·9 | 320 | 8 | 3·1 | 1440 | 14 | 14·0 |
| Total O ÷ total E | | | O/E = 0·55 | | | O/E = 2·57 | | | $P < 0·02$ | |

* Footnote and abbreviations as in Table b.


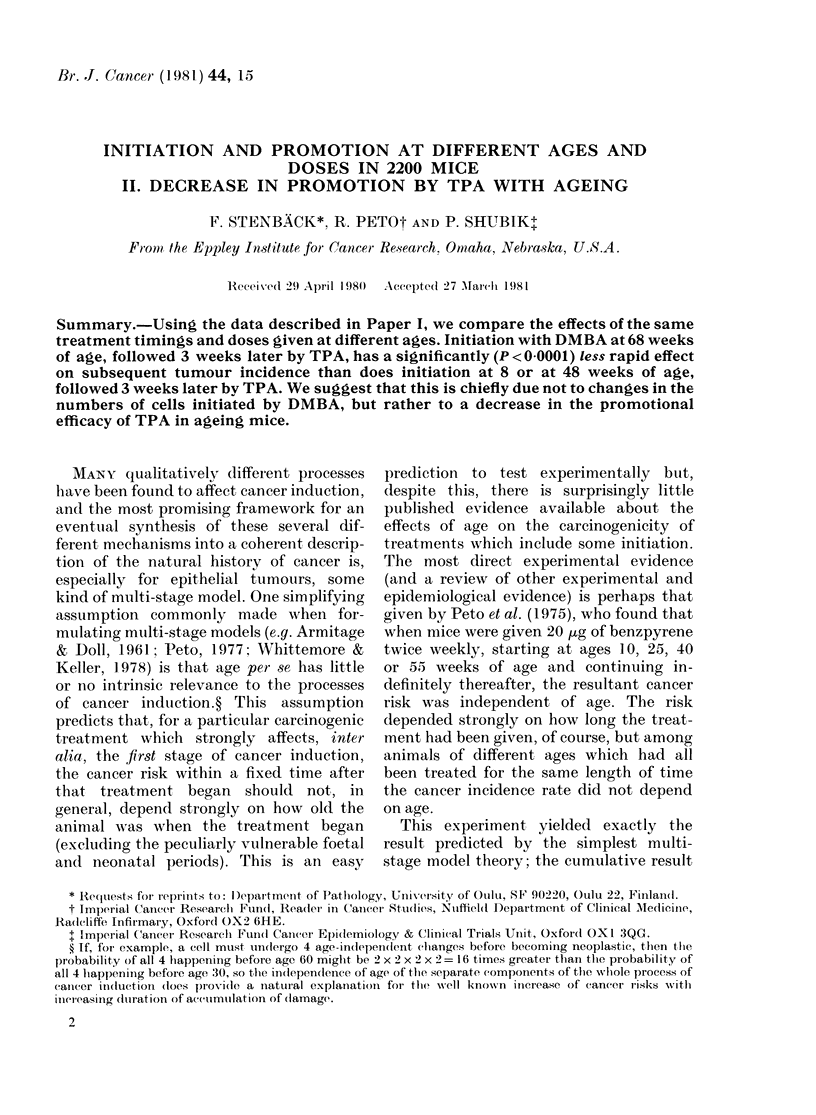

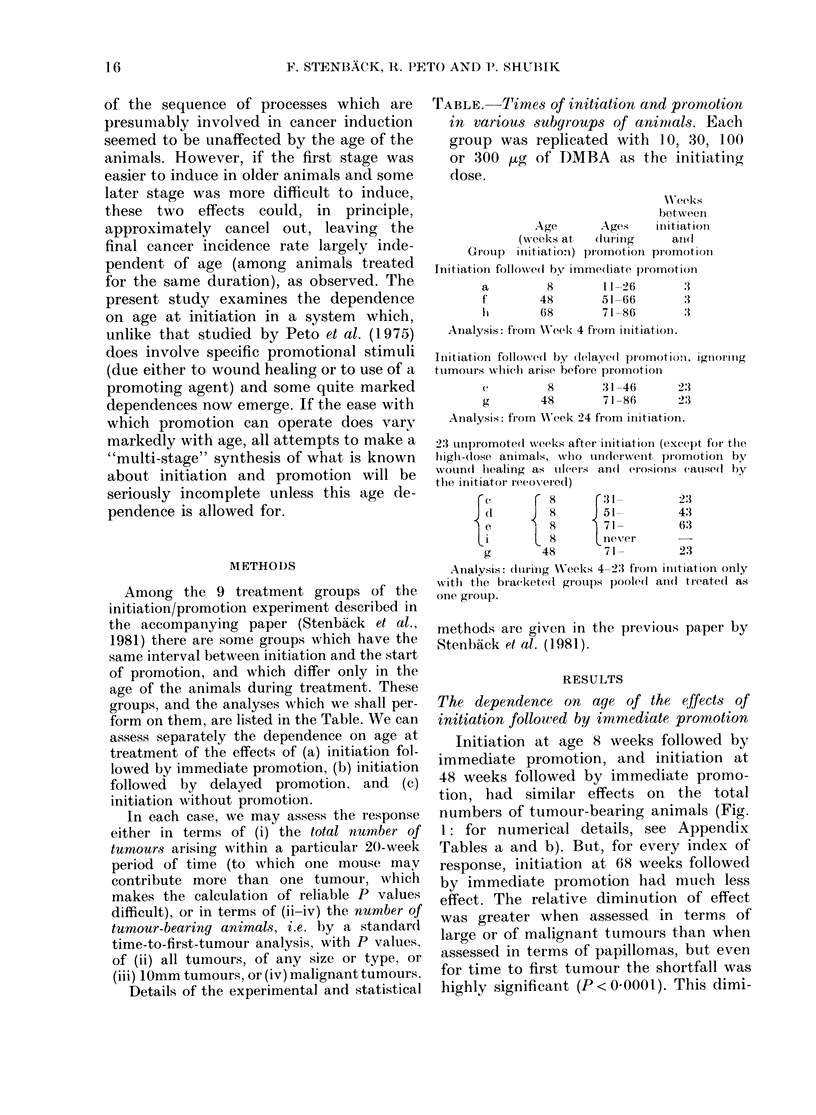

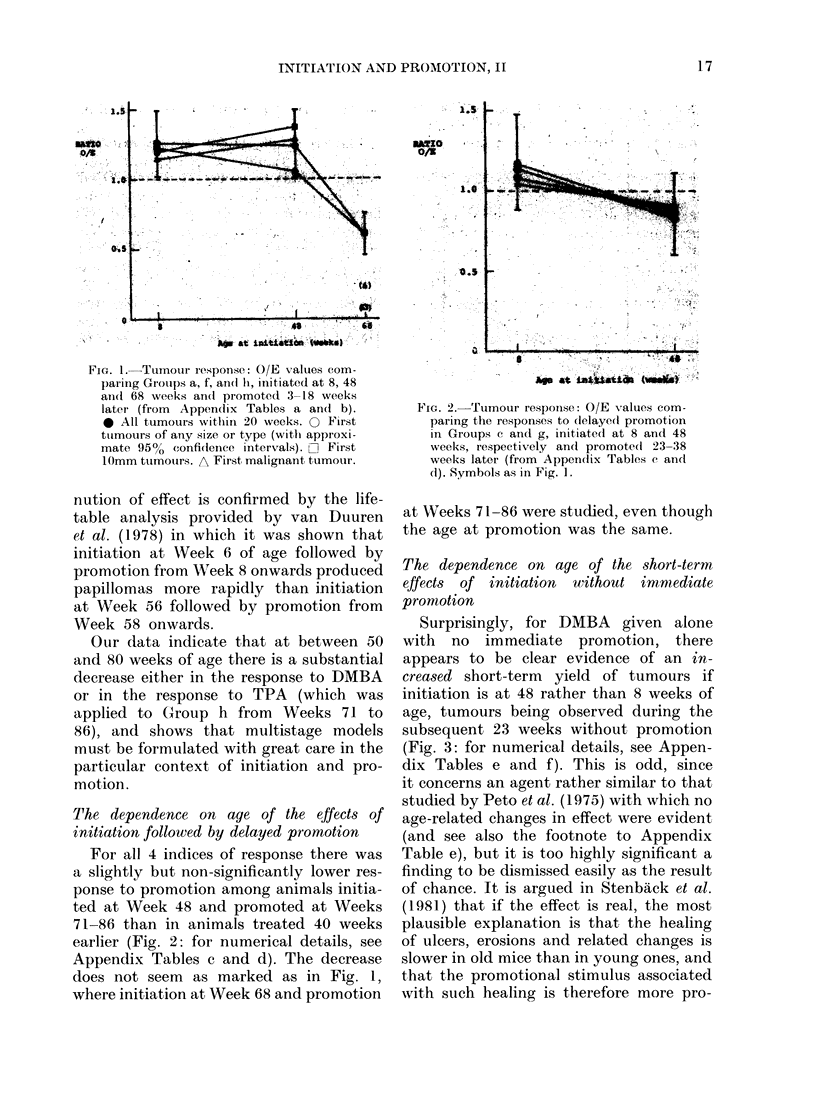

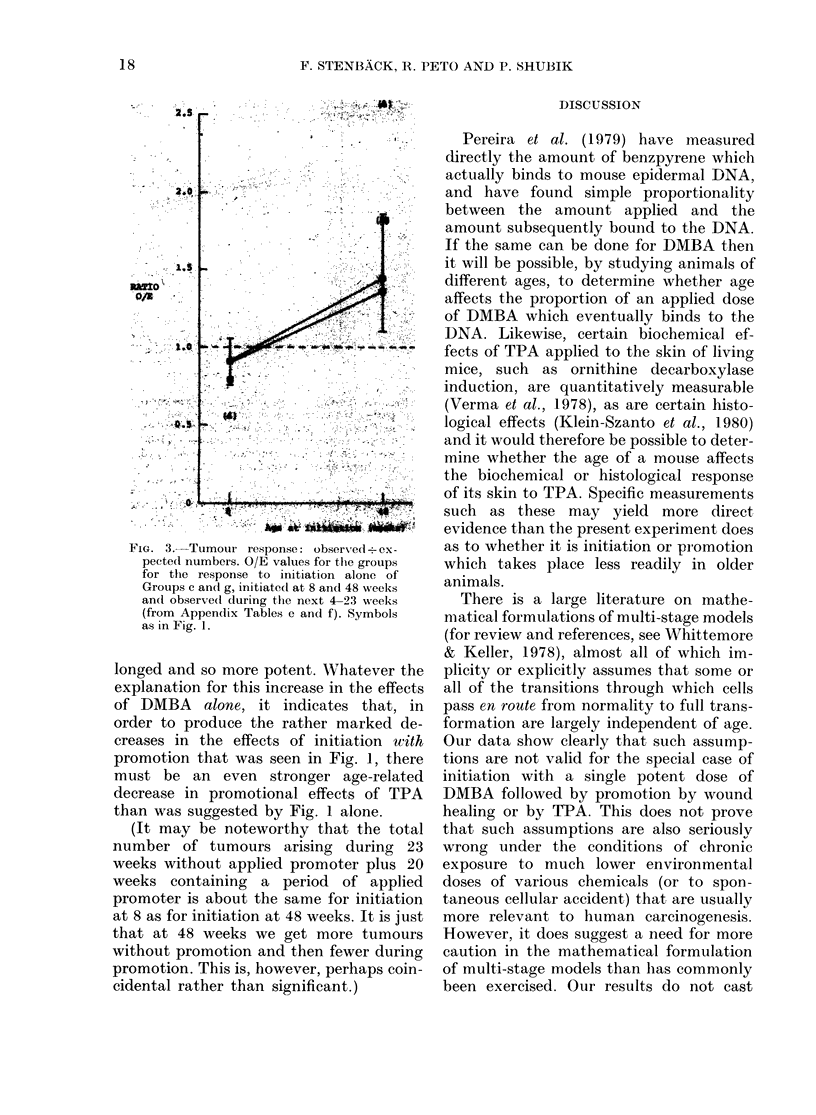

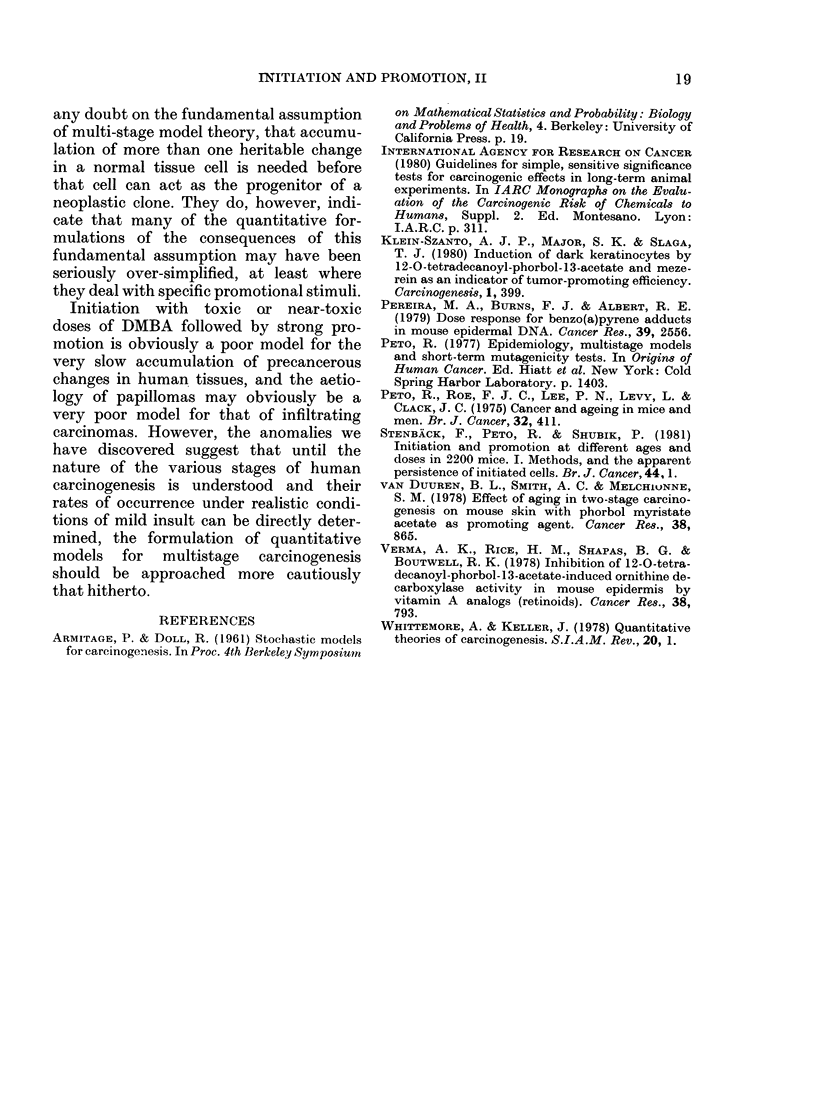

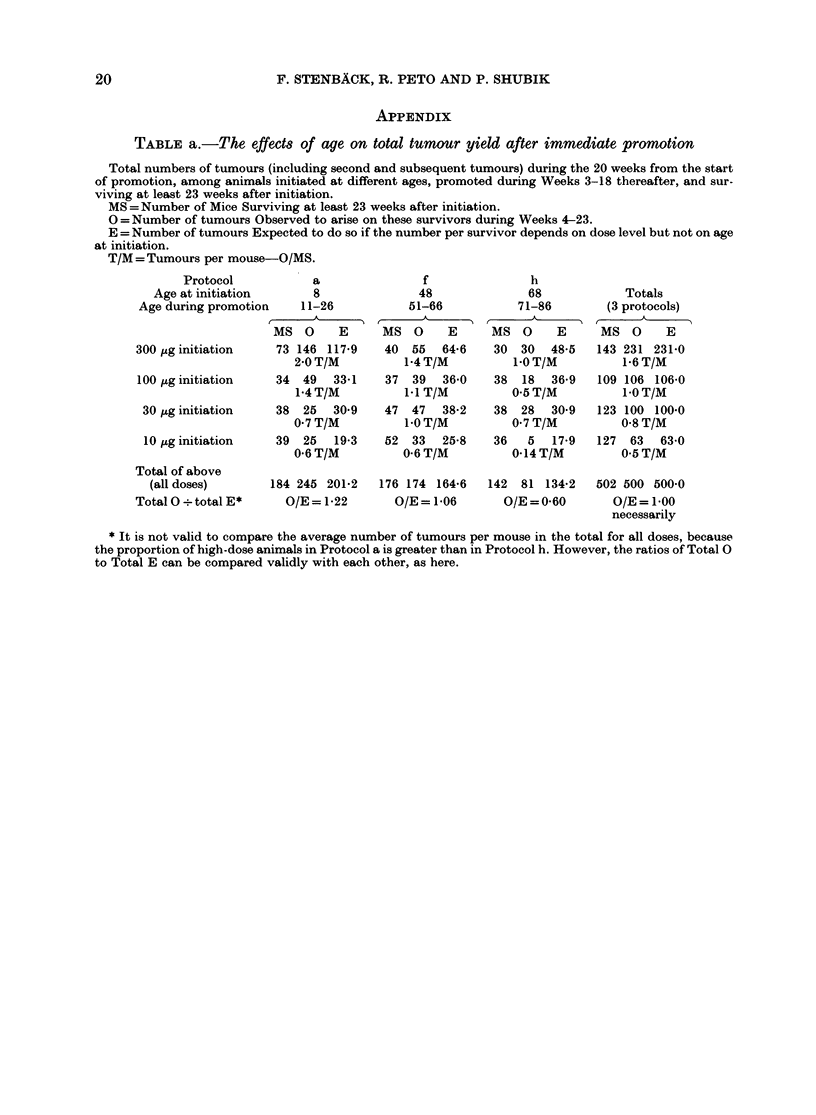

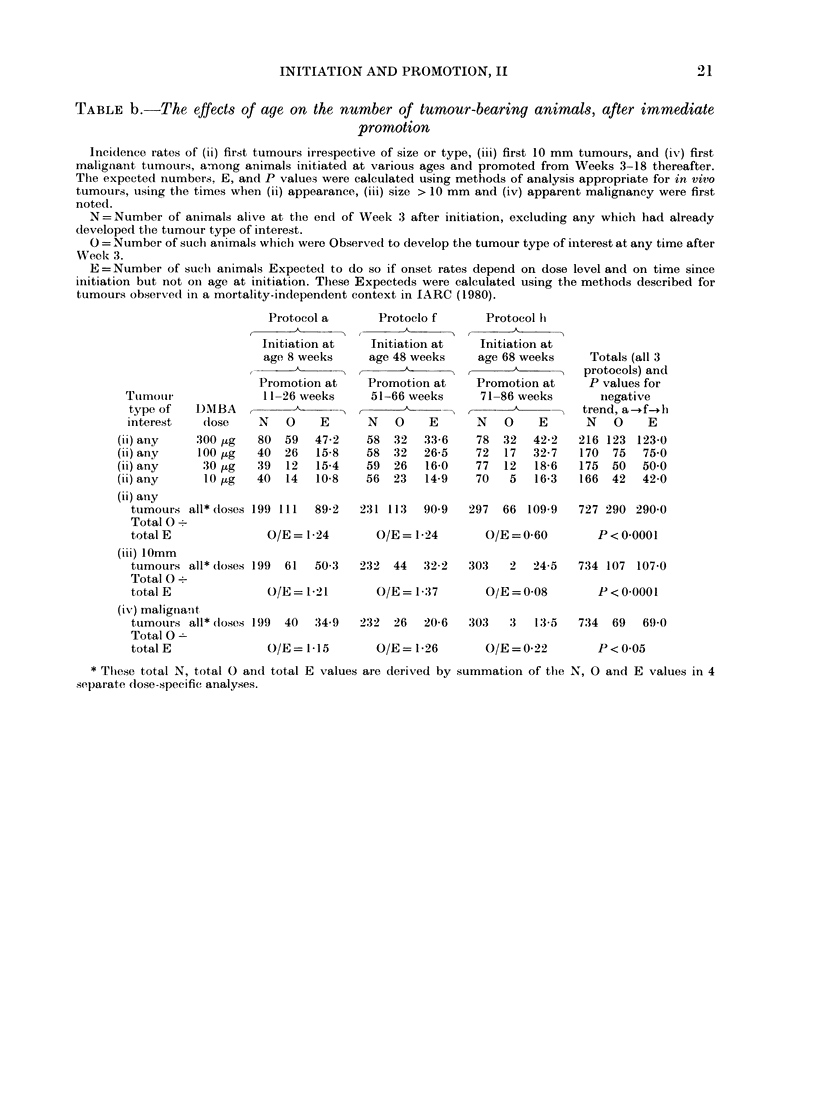

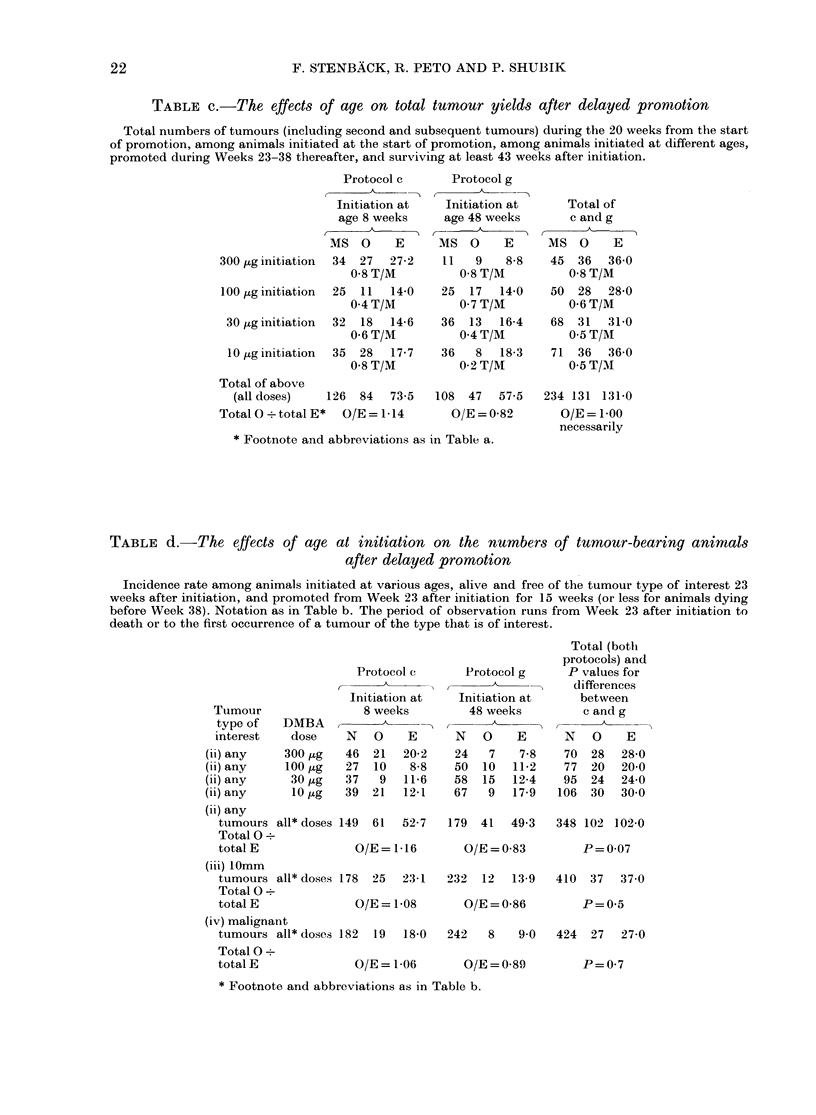

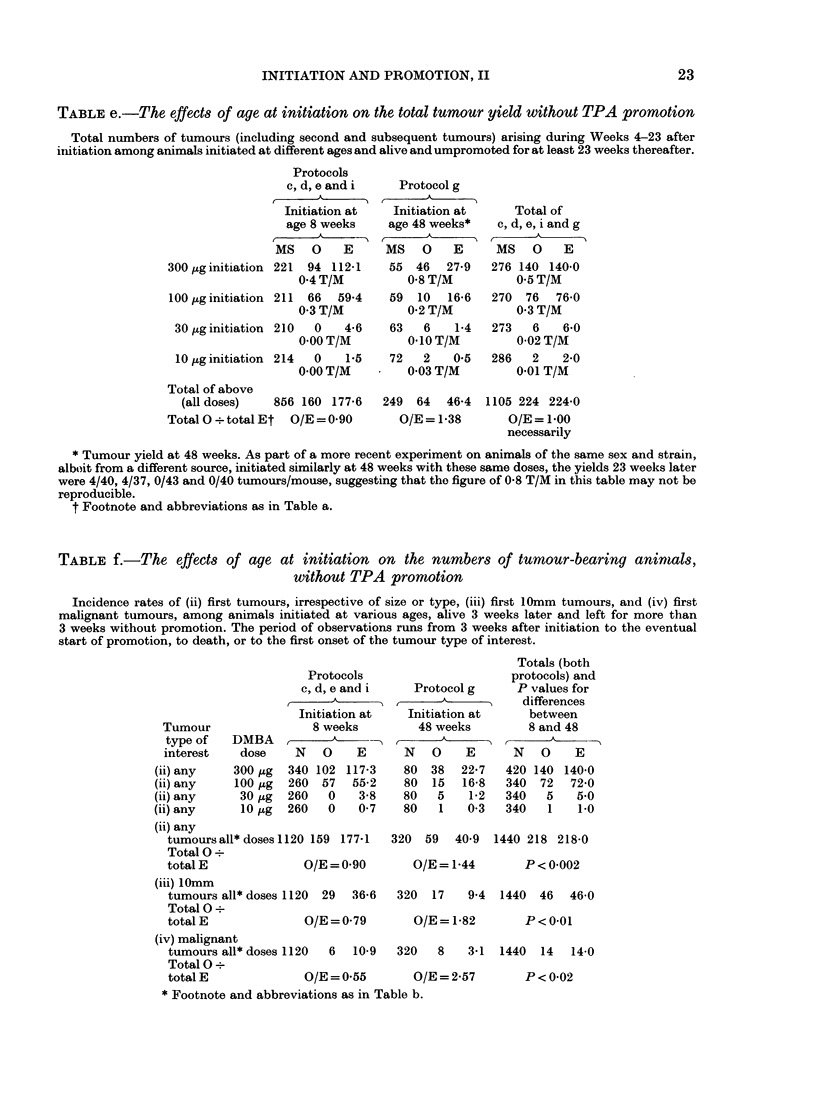


## References

[OCR_00504] Klein-Szanto A. J., Major S. K., Slaga T. J. (1980). Induction of dark keratinocytes by 12-O-tetradecanoylphorbol-13-acetate and mezerein as an indicator of tumor-promoting efficiency.. Carcinogenesis.

[OCR_00511] Pereira M. A., Burns F. J., Albert R. E. (1979). Dose response for benzo(a)pyrene adducts in mouse epidermal DNA.. Cancer Res.

[OCR_00521] Peto R., Roe F. J., Lee P. N., Levy L., Clack J. (1975). Cancer and ageing in mice and men.. Br J Cancer.

[OCR_00526] Stenbäck F., Peto R., Shubik P. (1981). Initiation and promotion at different ages and doses in 2200 mice. I. Methods, and the apparent persistence of initiated cells.. Br J Cancer.

[OCR_00532] Van Duuren B. L., Smith A. C., Melchionne S. M. (1978). Effect of aging in two-stage carcinogenesis on mouse skin with phorbol myristate acetate as promoting agent.. Cancer Res.

[OCR_00539] Verma A. K., Rice H. M., Shapas B. G., Boutwell R. K. (1978). Inhibition of 12-O-tetradecanoylphorbol-13-acetate-induced ornithine decarboxylase activity in mouse epidermis by vitamin A analogs (retinoids).. Cancer Res.

